# Synergistic Effects of Different Levels of Genomic Data for the Staging of Lung Adenocarcinoma: An Illustrative Study

**DOI:** 10.3390/genes12121872

**Published:** 2021-11-24

**Authors:** Yingxia Li, Ulrich Mansmann, Shangming Du, Roman Hornung

**Affiliations:** Institute of Medical Informatics, Biometry and Epidemiology, University of Munich, 81377 Munich, Germany; yingxiali@ibe.med.uni-muenchen.de (Y.L.); mansmann@ibe.med.uni-muenchen.de (U.M.); shangmingdu@ibe.med.uni-muenchen.de (S.D.)

**Keywords:** multi-omics data, lung adenocarcinoma, MKL, mRMR

## Abstract

Lung adenocarcinoma (LUAD) is a common and very lethal cancer. Accurate staging is a prerequisite for its effective diagnosis and treatment. Therefore, improving the accuracy of the stage prediction of LUAD patients is of great clinical relevance. Previous works have mainly focused on single genomic data information or a small number of different omics data types concurrently for generating predictive models. A few of them have considered multi-omics data from genome to proteome. We used a publicly available dataset to illustrate the potential of multi-omics data for stage prediction in LUAD. In particular, we investigated the roles of the specific omics data types in the prediction process. We used a self-developed method, Omics-MKL, for stage prediction that combines an existing feature ranking technique Minimum Redundancy and Maximum Relevance (mRMR), which avoids redundancy among the selected features, and multiple kernel learning (MKL), applying different kernels for different omics data types. Each of the considered omics data types individually provided useful prediction results. Moreover, using multi-omics data delivered notably better results than using single-omics data. Gene expression and methylation information seem to play vital roles in the staging of LUAD. The Omics-MKL method retained 70 features after the selection process. Of these, 21 (30%) were methylation features and 34 (48.57%) were gene expression features. Moreover, 18 (25.71%) of the selected features are known to be related to LUAD, and 29 (41.43%) to lung cancer in general. Using multi-omics data from genome to proteome for predicting the stage of LUAD seems promising because each omics data type may improve the accuracy of the predictions. Here, methylation and gene expression data may play particularly important roles.

## 1. Introduction

Lung cancer is one of the most common types of cancer. Morbidity and mortality associated with lung cancer rank high among all cancers worldwide [1]. Lung cancer can be divided into small cell lung cancer (SCLC) and non-small cell lung cancer (NSCLC). Lung adenocarcinoma (LUAD) is a histological subtype of NSCLC [2], accounting for approximately 70% of NSCLC cases. The five-year survival rate of LUAD remains very poor [3]. However, if diagnosed at an early stage, survival rates are greatly extended. One-year survival rates for stage I NSCLC are 81–85%, while a stage IV diagnosis is associated with only a 15–19% survival rate [4]. Due to the existence of different treatment methods, the staging of LUAD is an initial and important step in clinical diagnosis and targeted treatment. This highlights the need to design computational methods for the staging prediction of LUAD to reduce the overall mortality associated with this disease and further improve the quality of life of patients.

Since cancer is related to alterations in genes that control normal cell growth and death, molecular aberrations play a critical role in cancer initiation and progression [5]. An understanding of the molecular basis of cancer helps to predict the clinical outcome of cancer patients and determine the best-fitting treatments. With the development of sequencing technologies, data at the multi-molecular level have recently become increasingly available. Multi-omics genetic data frequently include copy number variation (CNV), methylation data, gene expression (RNA-seq), microRNA (miRNA), and protein expression from the same patients. Many researchers have attempted cancer classification using RNA-seq, miRNA, CNV, or DNA methylation [6,7,8]. For instance, Patnaik et al. used miRNA expression profiles to predict the recurrence of NSCLC [9]. Li et al. used an RNA-Seq dataset to perform prognosis and assess overall survival in lung cancer [10]. Jurmeister et al. performed DNA methylation analysis to distinguish between metastatic and primary lung cancer [11]. While such efforts are important, using only one factor can be expected to be associated with limited performance in prediction, as cancer is a phenotypic outcome of events accumulating through multi-omics dimensions from genome to proteome [12]. Genome variability, for example in CNV [13], can affect gene expression by altering gene dosage and regulating gene activity. Epigenetic changes, such as DNA methylation [14], affect gene expression by controlling the activity of genes. At the level of the transcriptome, gene expression and miRNA are the most representative data types. For example, miRNA can regulate every aspect of cellular activity [15]. At the proteomic level, proteins are undoubtedly the molecules associated the most with disease, and alterations in protein expression levels are directly related to disease [16].

Various machine learning methods, such as Random Forests (RF) [17], Bayes classifiers [18], and Support Vector Machines (SVM) [19] have been widely used to predict the clinical outcome of lung cancer based on genomic data. For example, Cai et al. [20] used multi-class SVM to classify lung cancer. Given the complexity and heterogeneity of the staging prediction of LUAD, more practical strategies were proposed. For instance, Li et al. [21] proposed predicting LUAD stages by combining SVM and RF. Dong et al. [22] used a multi-weighted gcForest method to integrate methylation data, RNA-seq, and CNV to predict the staging of LUAD. Many machine learning algorithms are also used in the prediction of clinical outcomes of different types of cancer by analyzing different genetic data types [23,24,25,26,27,28]. Recently, Hornung and Wright [29] designed a method called ‘block forests’ that modifies the split point selection of random forests to incorporate the group structure of multi-omics data. Apart from this specific method, there are several other multi-omics prediction methods [30,31,32,33,34] (see [29] for an overview)

In this paper, we aim to illustrate the value of multi-omics data from genome to proteome for the staging classification of LUAD. For the classification of multi-omics data, we use “Omics-MKL”, a self-developed algorithm which integrates filter-wrapper based feature selection and multiple kernel learning. Note that, since we analyze only a single dataset with limited sample size, we do not make any claims about the performance of Omics-MKL in comparison to other multi-omics prediction methods. We use Omics-MKL merely for illustrative purposes here, demonstrating that using multi-omics data for the staging of LUAD can lead to improved prediction performance in comparison to using single-omics data and that each omics data type adds to the performance of multi-omics prediction rules. We compare Omics-MKL with models that only use single-omics data, as well as with models that use multi-omics data, where for each of these models, one of the omics data types was removed. We also compared the method to basic machine learning methods.

## 2. Materials and Methods

### 2.1. Data Preparation

LUAD data were downloaded from the TCGA data portal (https://portal.gdc.cancer.gov/, accessed on 19 December 2019). TCGA [35] is a public database that contains thousands of cancer patient samples, different cancer types, and various omics data types. We selected multi-omics datasets, because we were interested in studying the impact of the fusion of different omics data types on LUAD cancer staging predictions. Among these types, CNV belongs to the genome level, methylation to the epigenetic level, gene expression and miRNA to the level of the transcriptome, and protein expression to the level of the proteome.

**Figure 1:**
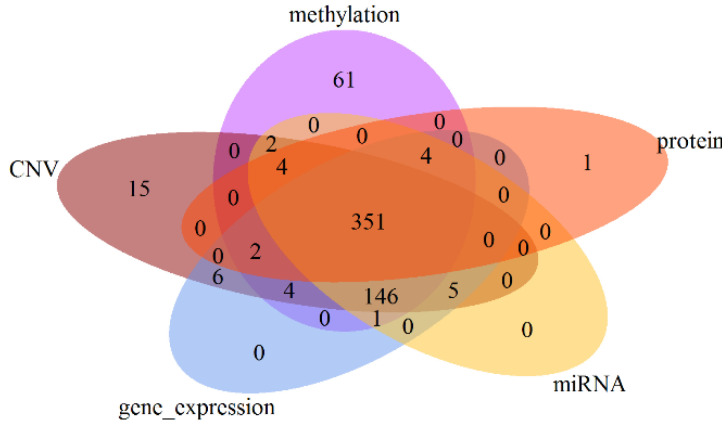
The case numbers available for each combination of omics data types.

We obtained 351 multi-omics data samples for analysis by first excluding samples without clinical staging information and then excluding samples that did not feature all considered multi-omics data types. Figure 1 shows the numbers of patients available for each possible combination of omics data types. In accordance with a previous study [36], we defined the staging prediction of LUAD in our study as a binary classification problem, differentiating between early stages (T1-T2, *n* = 270) and late stages (T3-T4, *n* = 81). Detailed information on the patients’ basic characteristics is given in Table 1.

We obtained 56,170 feature variables. Table 2 gives an overview of these variables. After feature selection, 70 features were retained. The feature selection process will be described in the following section.

### 2.2. Omics-MKL

#### 2.2.1. Feature Selection

To obtain a predictive subset of features and reduce the computational burden, we performed automatic feature selection. In general, feature selection methods can be categorized into filter methods, wrapper methods, and embedded methods [37]. In our article, we used a filter-wrapper method to select and model the features. The filter method sorts the features with respect to their importance and redundancy and the wrapper method aims at selecting a number of features that leads to the greatest classification performance.

##### Minimum Redundancy and Maximum Relevance (mRMR)

mRMR is a multivariate filter procedure that sorts features according to their predictive information, while taking into account their mutual information [38]. The aim of mRMR is to retain features that are maximally relevant for predicting the target class, but also minimally redundant among each other. It is a very popular method applied to select features in areas such as gene expression data analysis [39,40], protein sub-cellular localization prediction [41], and cancer survival prediction [42,43]. 

The mutual information between the jth  feature $x_{j}$ and the target class  c is defined in terms of the density functions $p\left(x_{j}\right)$, p(c), and $p\left(x_{j},c\right)$ as follows:

(1) $$I\left(x_{j};c\right)=\iint p\left(x_{j},c\right)log\frac{p\left(x_{j},c\right)}{p\left(x_{j}\right)p\left(c\right)}dx_{j}dc.$$

$I\left(x_{j};c\right)$ is a measure of relation between the individual feature $x_{j}$ and the target class c. For categorical features, the integrands in (1) reduce to sums and estimates for the involved density functions are readily available [38]. We transformed all continuous features into categorical features (see further down for details), because the mRMR implementation used in this paper requires categorical features (as do other implementations).

Let S denote a subset of features and |S|  the number of features in S. The Maximum-Relevance condition is:

(2) $$\max_{s}D\left(S,c\right),\;\;\;\;D\left(S,c\right)=\frac{1}{\left|S\right|}\sum_{x_{j}\in S}I\left(x_{j};c\right).$$

Although we can use the Maximum-Relevance algorithm to choose the top individual features in descending order of $I\left(x_{j};c\right)$, it has been recognized that the selected features could have rich redundancy, namely, “the m best features are not the best m features” [44]. To reduce the redundancy among selected features, a Minimum-Redundancy condition can be added:

(3) $$\min_{s}R\left(S\right),\;\;\;\;R\left(S\right)=\frac{1}{{\left|S\right|}^{2}}\sum_{x_{j},x_{j’}\in S}I\left(x_{j};x_{j^{\prime }}\right).$$

The mRMR feature set is obtained by optimizing the conditions in Equations (2) and (3) simultaneously.

In practice, a sequential incremental method is used. Suppose we already have $S_{m-1}$, a feature set with m−1 sorted features. Then the task is to select the mth feature, that is, the next sorted feature, from the set $\left\{X-S_{m-1}\right\}$, where set X represents the set of all features. This feature is selected by maximizing the single-variable relevance minus a redundancy function:

(4) $$max_{x_{j}\in X-S_{m-1}}\left[I\left(x_{j};c\right)-\frac{1}{m-1}\sum_{x_{j’}\in S_{m-1}}I\left(x_{j};x_{j^{\prime }}\right)\right].$$

The features are sorted using Formula (4), stopping at a maximum value of 500 sorted features.

As already noted above, mRMR requires the features to be categorical. Each continuous feature was, therefore, transformed into a categorical feature, which was performed as follows: the value −1 was assigned for feature values smaller than µ − ασ, the value 0 for feature values in [µ − ασ, µ + ασ], and the value 1 for feature values larger than µ + ασ. Here, µ is the mean of the values of the feature, σ is their standard deviation, and α is a parameter controlling the expression rate, which was set to 0.5.

##### Feature Selection Process Based on the Filter-Wrapper Method

The filtering step using mRMR does not yet deliver a compact set of selected features, but rather a list of 500 sorted features. These 500 features do, however, likely not deliver an optimal prediction rule with respect to the classification method we use. To tackle this issue, we used a filter-wrapper method to select features.

A wrapper [45] method can convolve with a classifier and has the direct goal of maximizing the prediction performance of a particular classifier. After obtaining a sorted list of 500 features using mRMR, we applied the following wrapper method: first, for m = 20, 30, 40, …, 500, apply the considered classification method (see next subsection) using only the first m features and calculate the cross-validated AUC value associated with the resulting prediction rule. Second, identify the optimal number N of first genes as that number of genes that was associated with the largest cross-validated AUC value in the first step.

#### 2.2.2. Multiple Kernel Learning Classification

##### The General MKL Model

In our study, we combined different data types into one model. Different omics data types have different feature representations, which is why directly combining these multiple sources of data as an input of one model would not be efficient [46]. Multiple Kernel Learning (MKL) can fuse heterogeneous omics data by using different kernels to represent input from different sources. 

Equation (5) combines *M* kernels to one single kernel in a linear format:

(5) $$K\left(x,x^{\prime }\right)=\overset{M}{\underset{m=1}{\sum }}d_{m}K_{m}\left(x,x^{\prime }\right),\;with\;d_{m}\geq 0,\;\overset{M}{\underset{m=1}{\sum }}d_{m}=1$$

where x and $x^{\prime }$ both represent a vector of all features, $K_{m}\left(x,x^{\prime }\right)$ indicates the mth kernels, and $d_{m}$ is the weight of the mth kernel. Note that it is not only possible to use different kernels for different data types, but there can also be several kernels for the same data types.

Bach et al. [47] have shown that the MKL formulation is actually a dual SVM problem. The approach simpleMKL is a supervised method based on an improvement of the linear MKL framework, the decision boundary of which is given by:

(6) $$f\left(x\right)=\overset{l}{\underset{i=1}{\sum }}a_{i}^{*}K\left(x,x_{i}\right)+b^{*}\;$$

where *l* is the number of patients and $x_{i}$ denotes the vector of all features for the *i*th patient. When applying the classifier to the feature vector of a new patient, the sign of f(x) is used to decide on which of the two classes the patient is classified into. To optimize the two parameters of the SVM and the kernel coefficients, simpleMKL uses an iterative gradient descent method. This approach has proven to be efficient when the number of kernels is high [48]. Importantly, the particular MKL implementation considered in this paper uses an $L_{2}-\mathrm{norm}$ regularization leading to a sparse solution in the kernel coefficients. The optimization problem is of the form:

(7) $$min_{f,b,\varepsilon }\;\frac{1}{2}{||f||}_{H}^{2}+C\sum_{i}\varepsilon_{i}\;s.t.\;\;y_{i}\left(f\left(x_{i}\right)+b\right)\geq 1-\varepsilon_{i}\;\forall_{i}\;\varepsilon_{i}\geq 0\;\forall_{i}$$

where ${||f||}_{H}$ denotes a kernel in Hilbert space associated with a kernel $K_{m}$ and $y_{i}$ denotes the outcome.

The overall kernel can be divided into the individual kernels, replacing ${||f||}_{H}$ by $\underset{m}{\sum }||f_{m}||{}_{Hm}$, which leads to:

(8) $$min_{\left\{f_{m}\right\},b,\varepsilon ,d}\;\frac{1}{2}\sum_{m}||f_{m}||{}_{Hm}^{2}+C\sum_{i}\varepsilon_{i}\;s.t.y_{i}\left(\sum_{m}f_{m}\left(x_{i}\right)+y_{i}b\right)\geq 1-\varepsilon_{i}\;\;\forall_{i}\;\varepsilon_{i}\geq 0\;\;\forall_{i}\;\sum_{m}d_{m}=1,\;d_{m}\geq 0\;\;\forall_{m}$$

This equation shows several kernels in Hilbert space being combined in $L_{2}-\mathrm{norm}$ formation. Detailed information can be found in [49].

##### The MKL Model for Multi-Omics Data

MKL can fuse heterogeneous omics data by employing different kernels for the different omics data types and also several kernels per data type in an effort to make the decision function more powerful and improve the prediction performance. Therefore, in Omics-MKL, we use the simpleMKL method to construct different independent kernels for different omics data types, integrating them into a universal model. Specifically, we construct 10 different kernels for the five considered omics data types (CNV, gene methylation, gene expression, miRNA, and protein). For the kernels, we use two types of kernel functions for each omics data type, the Gaussian kernel and the polynomial kernel. As seen in the previous subsection, the simpleMKL method directly solves an integrated support vector machine optimization problem instead of learning kernel combinations from independent kernels, which greatly reduces the computational cost [43].

### 2.3. Experimental Design

To evaluate the performance of the methods, we used 10-fold nested cross-validation [50]. Nested cross-validation includes an outer loop and an inner loop. In the inner loop, we determine an optimal number of features N out of 20, 30, 40, …, 500, where we use mRMR to sort the features. In the outer loop, the best N from the inner loop is carried forward to build the final model and the performance is evaluated. The workflow is visualized in Supplementary Figure S1. Note that we applied the filter-wrapper approach using mRMR for all compared methods.

We used the receiver operating characteristic (ROC) curve and the area beneath it, the AUC, to evaluate the performance of the algorithms.

All analyses were performed in MATLAB R2020b. The code is available on GitHub (https://github.com/yingxiali/Omics_MKL, accessed on 19 November 2021).

## 3. Results

### 3.1. Comparison of the Achieved Prediction Performances When Using Multi-Omics Data and Single-Omics Data

To assess the added value of multi-omics data over single-omics data, we compared the classification performances of Omics-MKL when using single-omics data (methylation, CNV, miRNA, RNA-seq, protein) and multi-omics data. Specifically, six different Omics-MKL-based prediction rules were constructed, each data type using two kernel shapes.

**Figure 2:**
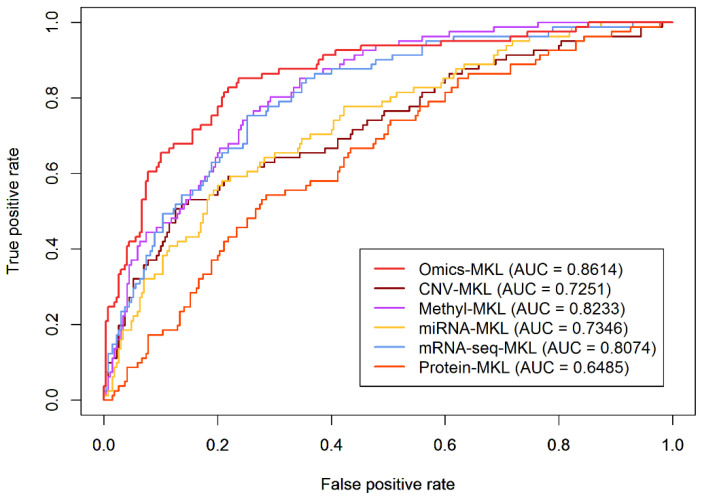
Comparison between prediction performance obtained using multi-omics data and single-omics data (prediction method: Omics-MKL).

As Figure 2 shows, among the single-omics prediction rules, the one using methylation data (Methyl-MKL) has the highest AUC value of 0.8233, and gene expression data (RNA-seq-MKL) showed comparable performance with an AUC of 0.8074. However, none of the AUC values obtained based on the single-omics data sources were larger than the AUC of 0.8614 obtained when considering all omics data types concurrently (Omics-MKL). This illustrates that integrating multi-omics genetic data can effectively improve the accuracy of LUAD staging compared to using only single-omics data.

### 3.2. Effectiveness of Integrating Multiple Omics Data Types 

**Figure 3:**
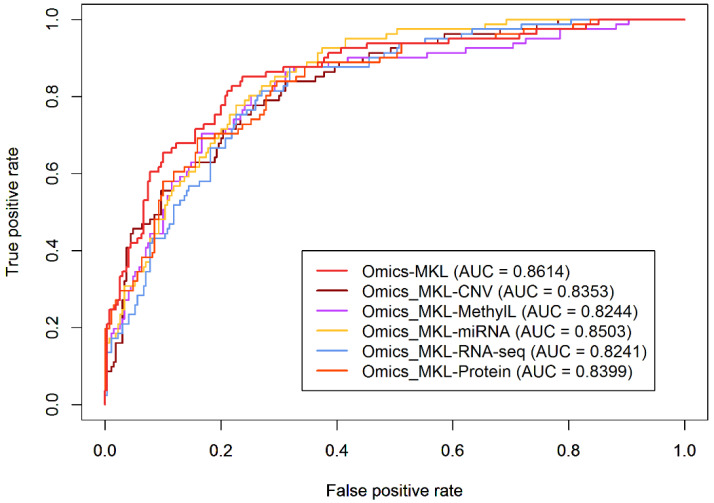
Comparison between the prediction performance obtained using all five omics data types and after removing one omics data type at a time (prediction method: Omics-MKL).

In this subsection, we illustrate that each considered omics data type can contribute to an improved prediction performance when using multi-omics data. In an iterative fashion, we individually removed methylation, CNV, miRNA, RNA-seq, or protein information and considered an Omics-MKL prediction rule without the removed data type. This also allows for understanding about which omics data types play important roles in prediction. The smaller the AUC becomes after removing an omics data type, the more important the respective data type tends to be. As seen in Figure 3, the prediction rule based on all available omics data types performed best, suggesting that each data type improves the prediction of LUAD staging. Gene expression and methylation seem to play more important roles than the other data types. After removing the methylation and RNA-seq data from the multi-omics data, the AUC decreased by 0.0397 and 0.0400, respectively, whereas after removing the miRNA data, the AUC only decreased by 0.0138. The fact that the AUC decreased after removing each of the omics data types suggests that the integration of all available genomic data sources can be beneficial in terms of prediction performance in the staging of LUAD.

### 3.3. Comparison with Basic Machine Learning Methods Using Multi-Omics Data

**Figure 4:**
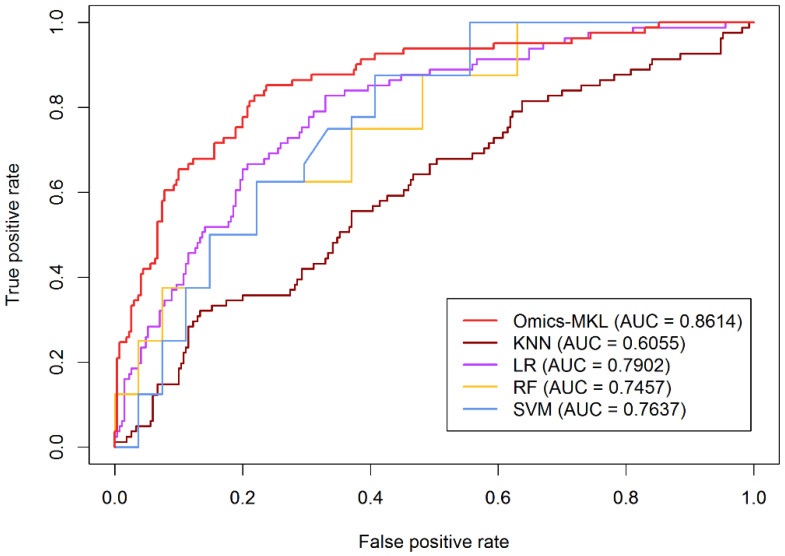
Comparison of the prediction methods applied to the whole multi-omics dataset.

As already discussed in the introduction, we do not make any claims on the effectiveness of Omics-MKL over that of other multi-omics prediction methods. However, to exclude that Omics-MKL does not deliver meaningful predictions, we compare it with basic machine learning algorithms, namely SVM, K-nearest neighbors (KNN), logistic regression (LR), and random forests (RF). The results are shown in Figure 4. The Omics-MKL algorithm delivered the largest AUC value. More precisely, Omics-MKL delivered an AUC value of 0.8614, which is 25.59%, 11.57%, 9.77%, and 7.12% higher than that obtained for KNN, RF, SVM, and LR, respectively. 

### 3.4. Analysis of the Selected Features

In the previous subsections, we performed nested cross-validation to evaluate the performance of the compared approaches. With this procedure, in each iteration of the outer cross-validation loop, a different subset of omics features is selected. However, it would be interesting to obtain a single set of selected features to investigate which features seem to be particularly important for stage prediction using multi-omics data in LUAD. To obtain such a single set of selected features, we first performed a non-nested 10-fold cross-validation for each considered N value (20, 30, …, 500), repeating mRMR in each iteration. Subsequently, we used the N value that was associated with the maximum cross-validated AUC value to perform the final feature selection using the whole dataset, that is, without cross-validation.

**Figure 5:**
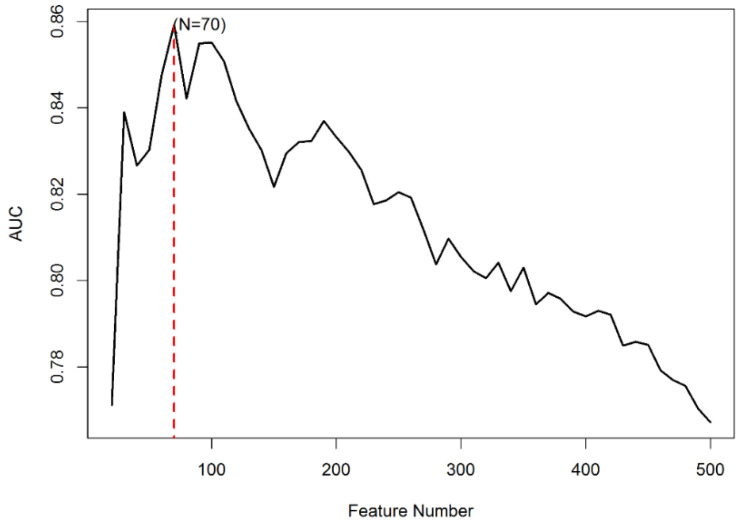
The relationship between the number of selected features and the cross-validated AUC.

**Figure 6:**
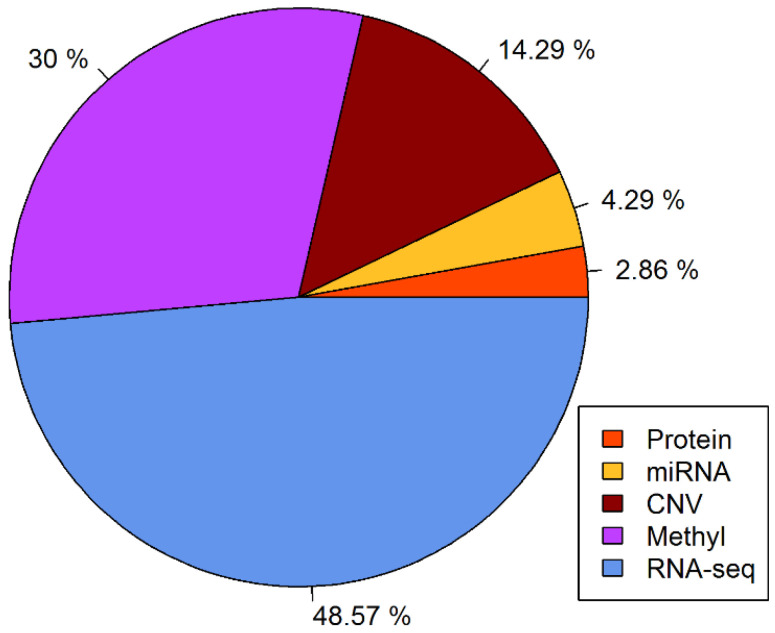
The percentages of features from each data type in the selected features.

Figure 5 illustrates that, when the value of N is varied, the performance of the model changes strongly. For N equal to 70, the cross-validated AUC value of the Omics-MKL classifier was best. We provide the cross-validated AUC values for N = 20, 30, …, 70 in Supplementary Table S1. The percentages of the different data types among the selected features are shown in Figure 6. The selected features included 10 (14.29%) CNV features, 21 (30%) methylation features, 34 (48.57%) gene expression features, 3 (4.29%) miRNA features, and 2 (2.86%) protein expression features (cf. also Table 2).

**Figure 7:**
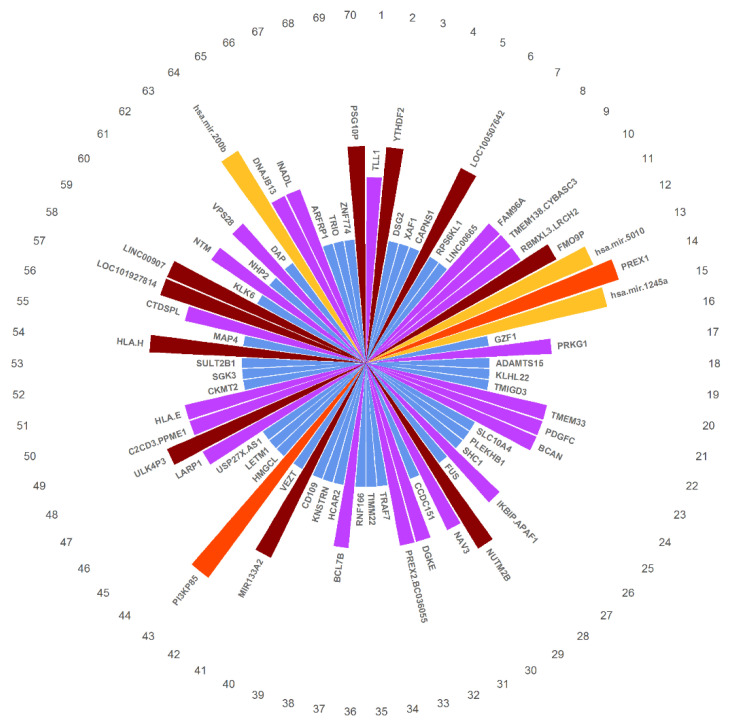
The selected features ranked according to their association with the outcome and the mutual information between them. Red represents protein features, yellow represents miRNA features, brown represents CNV features, purple represents methylation features, and blue represents RNA-seq features.

Because we applied mRMR before feature selection, the selected features are sorted according to their association with the outcome and the mutual information between them. The ranks of the selected features are shown in Figure 7.

### 3.5. Enrichment Analysis of the Selected Features

**Figure 8:**
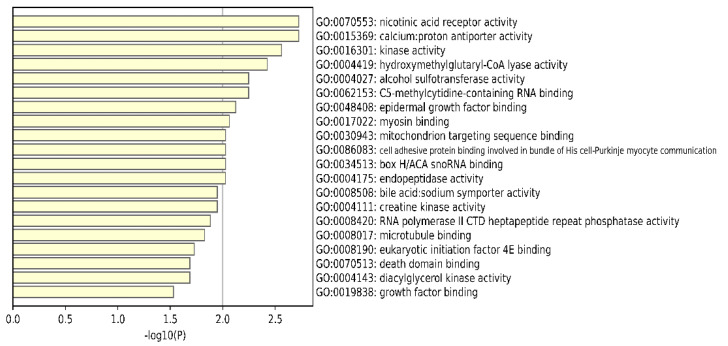
Bar graph of enriched molecular functions based on the 70 selected features.

To further understand the roles of the selected features, we conducted an enrichment analysis of these features. Using Metascape [51], to understand the differences between LUAD stages, the whole set of human genes was employed as the background against the GO and the Kyoto Encyclopedia of Genes and Genomes (KEGG) Pathway databases. The resulting molecular functions (MFs) are shown in Figure 8. We can see that the enriched functions were kinase activity and RNA expression-related functions. In addition, the most significantly enriched biological processes (BPs), cellular components (CCs), and KEGG pathways were negative regulation of catabolic processes, the centriolar satellite, and the Phospholipase D signaling pathway (see Supplementary Figures S2–S4).

### 3.6. Analysis of Those Selected Features That Are Known to Be Associated with LUAD

We searched all 70 selected features on the NCBI database and found that 18 of these features were reported to have functions in LUAD. Moreover, 11 features have been reported to be associated with lung cancer progress before. Table 3 lists the top ten features related to LUAD ranked by mRMR. In addition, we provide the reported information on the remaining selected features in Supplementary Table S2. 

*DSG2* gene overexpression has been found to correlate with poor prognosis in LUAD patients [52]. The *XAF1* gene has been found to inhibit cell proliferation and induce apoptosis in human LUAD cell line A549 in vitro [53]. The *CAPN1* gene has been proven to promote malignant behavior and erlotinib resistance mediated in LUAD [54].

**Figure 9:**
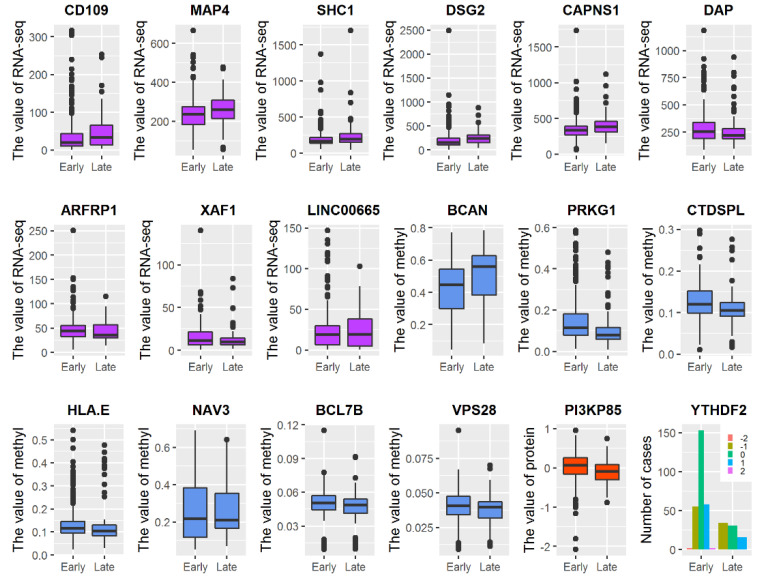
Relation between the values of the 18 selected features known to be associated with LUAD and the stage. Purple boxes represent RNA-seq features, blue boxes DNA methylation features, red boxes protein features, and bars CNV features (CNV values: −2 = homozygous deletion; −1 = hemizygous deletion; 0 = neutral/no change; 1 = gain; 2 = high level amplification).

To further understand the differences between LUAD stages, we performed a statistical analysis of the 18 selected genes known to be associated with LUAD. As seen in Figure 9, for RNA-seq data, the expression of *CD109*, *MAP4*, *SHC1*, *DSG2*, and *CAPNS1* in the early stage of LUAD was lower than that in the late stage of LUAD, while the expression of *DAP*, *ARFRP1*, and *XAF1* was lower in the late stage of LUAD. In the case of the methylation data, only *BCAN* had lower methylation at early stages, while *PRKG1*, *CTDSPL,* and *HLA.E* had lower methylation at late stages. Among the 18 LUAD-related features, only one protein feature was selected, *PI3KP85*, which was expressed more strongly in the early stages of LUAD. Interestingly, for the selected CNV feature YTHDF2, there was no change in most patients in the early stages and no homozygous deletion and high-level amplification for any of the patients in the late stages.

## 4. Discussion 

Using five omics data types jointly delivered better classification performance than when using only four omics data types or single-omics data. These results indicate that combining various omics data types into multi-omics data seems to be an efficient way of improving the classification of lung adenocarcinoma staging. 

We used the self-developed method Omics-MKL in our experiments. Given that we did not compare Omics-MKL to other multi-omics prediction approaches and that we only analyzed one specific dataset, it is not possible to recommend Omics-MKL without limitation in clinical applications. Other multi-omics approaches may deliver better prediction results.

The focus in this paper was not on Omics-MKL, but on illustrating the predictive value of multi-omics data in the staging of LUAD. An advantage of Omics-MKL in the context of the analyses performed in this paper was that the method functions in the same way when applied to single-omics data as when applied to multi-omics data. This makes the results obtained for multi-omics data and single-omics data comparable. In contrast, if we had used different methods for multi-omics data and single-omics data, this would have hampered the comparability between the results obtained for these two data types. Omics-MKL performed superior to the considered traditional machine learning classification methods for the investigated dataset. A possible reason for this is that, by using different kernels for different omics data types, Omics-MKL may better capture heterogeneous information from different types of data than the other compared methods, which do not explicitly consider that the features stem from different omics data types. An advantage of using mRMR for multi-omics data is that, by minimizing redundancy in the feature selection, we account for the known fact that the predictive information in different omics data types overlaps strongly.

Gene expression and methylation features were the two most important omics data types in our experiments. Methylation data played the most important role when building LUAD staging models using single-omics data. DNA methylation alteration is frequently observed in LUAD and plays an important role in carcinogenesis, diagnosis, and prediction [55,56]. The promoter regions of tumor suppressor genes are often hypermethylated, resulting in the activation of corresponding genes in tumors. It has been reported that *BRCA2* [57], *BCL2* [58], *APC* [59], and *p16* [60] are hypermethylated in NSCLC, and *P16* [60] gene promoter methylation is used as a biomarker for the diagnosis of NSCLC.

Our study has several limitations. First, the sample size for the multi-omics data is relatively small, which is why the performance estimates are likely quite variable. As shown in [61], it is not possible to quantify unbiasedly the variability of cross-validated performance estimates, which is why we are not able to investigate whether the observed performance differences between the methods are statistically significant. Second, our experiments integrated only omics data. Clinical information and pathological images were not considered in our study. Third, this work considered only internal validation via cross-validation. To obtain definitive conclusions on the ranking between the approaches, it would be necessary to analyze large numbers of multi-omics data samples, which are not available at this point. Moreover, it would also be interesting to compare the investigated methods using external validation. Including clinical information and imaging data would likely improve the performance further in comparison to using multi-omics data alone. In future work, we also intend to consider classifying cancer subtypes.

## 5. Conclusions

In this article, we used a self-developed method, Omics-MKL, for evaluating and illustrating the predictive value of multi-omics data in the staging of LUAD based on a publicly available dataset. Our results clearly indicate that using multi-omics data for the staging of LUAD has the potential to outperform using single-omics data and that each omics data type improves the predictions. At the same time, through the analysis of important genes and pathways, we tried to find some biological explanations for the differences between LUAD stages, and provide guidance for exploring the biological models of these stages.

## Figures and Tables

**Table 1:** Patients’ basic characteristics.

Items		Number	Percent (%)
Gender	Male Female	162 189	46.15 53.85
Age (year)	<60 ≥60 Unknown	94 237 20	26.78 67.52 5.70
Average age at diagnosis	65.04		
Stage	Early (T1, T2) Late (T3, T4)	270 81	76.92 23.08
Total		351	

**Table 2:** Data description.

Data Type	No. of Features	No. of Selected Features
CNV	25,988	10
Methylation	13,620	21
Gene Expression	15,751	34
miRNA Expression	595	3
Protein Expression	216	2
Total	56,170	70

**Table 3:** The top 10 features related to LUAD.

Rank ID	Genes	The Content of the Report	PubMed ID
2	YTHDF2	The m6A-related genes METTL3, YTHDF1, and YTHDF2 could serve as novel biomarkers for the prognosis of LUAD.	PMID: 32086933
3	DSG2	High DSG2 expression in both lung adenocarcinoma (LUAD) cell lines and tissues is associated with poor prognosis in LUAD patients.	PMID: 32272148
4	XAF1	XAF1 inhibits cell proliferation and induces apoptosis in the human lung.	PMID: 25539606
5	CAPN1	CAPN1 promotes malignant behavior and erlotinib resistance mediated by phosphorylation of c-Met and PIK3R2 via degrading PTPN1 in lung adenocarcinoma.	PMID: 32395869
8	LINC00665	Long non-coding RNA LINC00665 promotes lung adenocarcinoma progression and functions as ceRNA to regulate AKR1B10-ERK signaling by sponging miR-98.	PMID: 30692511
17	PRKG1	The MAPK, PI3K-Akt, Ras, and cGMP-PRKG1 signaling pathways were considered to be most probably correlated with platinum resistance.	PMID: 29288364
23	BCAN	A survival prediction model composed of six TME-related genes (CLEC17A, TAGAP, ABCC8, BCAN, FLT3, and CCR2) was used in a Lung Adenocarcinoma Microenvironment.	PMID: 32337264
26	SHC1	In NSCLC, the failure of pathways which involve factors such as DAPK1, GADD45A, SHC1, and TP53, in response to short telomeres, could promote tumor progression.	PMID: 22433385
30	NAV3	The most commonly mutated genes with predicted neo-antigens are KRAS, TTN, RYR2, MUC16, TP53, USH2A, ZFHX4, KEAP1, STK11, FAT3, NAV3, and EGFR in lung adenocarcinoma.	PMID: 30075702
37	BCL7B	Compared with the combined human ACs, 39 genes with similar expression changes in murine lung tumors and human ACs/LCCs were identified, such as the oncogene related BCL7B, the cell cycle regulator CDK4, and the proapoptotic Endophilin B1.	PMID: 14647414

## Supplementary Materials

The following are available online at https://www.mdpi.com/article/10.3390/genes12121872/s1, Figure S1: 10-fold Nested Cross-Validation with Omics_MKL, Figure S2: Bar graph of enriched biological processes based on the 70 selected features, Figure S3: Bar graph of enriched cellular components based on the 70 selected features, Figure S4: Bar graph of enriched KEGG pathways based on the 70 selected features, Table S1: Cross-validated AUC values for N = 20 to 70, Table S2: The reported information on the remaining selected features.
