# Supplementary material for: Synergistic Effects of Different Levels of Genomic Data for the Staging of Lung Adenocarcinoma: An Illustrative Study
Source: Genes (Basel). 2021 Nov 24;12(12):1872. doi: 10.3390/genes12121872 (PMC8700916; doi:10.3390/genes12121872)
Supplement: Supplementary file 1 [file genes-12-01872-s001.zip › supplementary Figure S1.pdf]

```
For i = 1 to 10 splits do
    Split D into D_i^train, D_i^test for ith split
    For n = 20, 30, 40, …, 500 do
        For j = 1 to 10 splits do
            Split D_i^train into SubD_j^train, SubD_j^test
            Index_n <-  mrmr (SubD_j^train, n)
            Sub_n_ D_j^train <- SubD_j^train(Index_n)
            Sub_n_ D_j^test <- SubD_j^test(Index_n)
            Predict_j <- simpleMKL (Sub_n_ D_j^train, Sub_n_ D_j^test)
            Auc_j <- auc (Predict_j)
        End
        Auc_n <- mean(Auc_j)
    End
    [best_Auc, best_N] = max(Auc_n)
    Index_N <-  mrmr (D_i^train, best_N)
    Sub_N_ D_i^train <- D_i^train(Index_N)
    Sub_N_ D_i^test <- D_i^test(Index_N)
    Predict_i <- simpleMKL (Sub_N_ D_i^train, Sub_N_ D_i^test)
    AUC_i <- auc (Predict_i)
End
AUC <- mean(AUC_i)
```

Figure S1. 10-fold Nested Cross-Validation with Omics_MKL

Note: a. D represents all omics data. n is the number of selected features.

b. mrmr is a filter method of feature selection.

(mrmr: Minimum Redundancy and Maximum Relevance)

c: simpleMKL: is a classifier.

(MKL: multiple kernel learning)
