# Supplementary figures and images for: Synergistic Effects of Different Levels of Genomic Data for the Staging of Lung Adenocarcinoma: An Illustrative Study
Source: Genes (Basel). 2021 Nov 24;12(12):1872. doi: 10.3390/genes12121872 (PMC8700916; doi:10.3390/genes12121872)

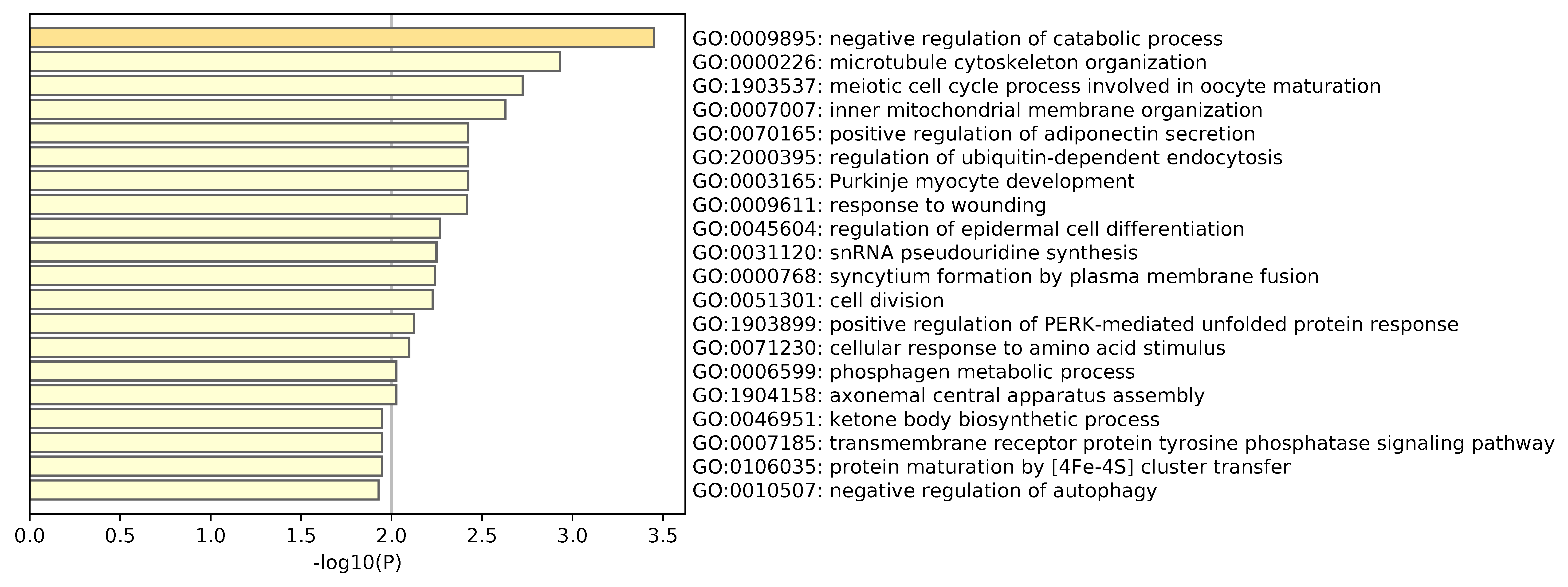

Supplement: Supplementary file 1 [file genes-12-01872-s001.zip › supplementary Figure S2.tiff]

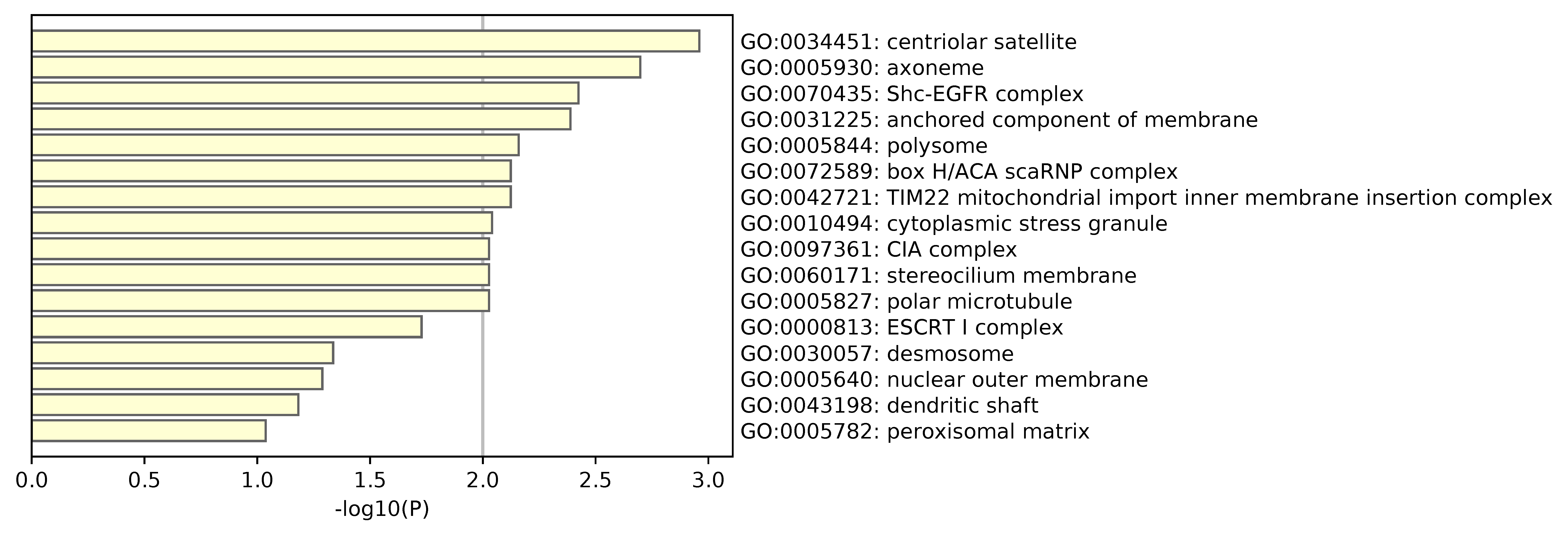

Supplement: Supplementary file 1 [file genes-12-01872-s001.zip › supplementary Figure S3.tif]

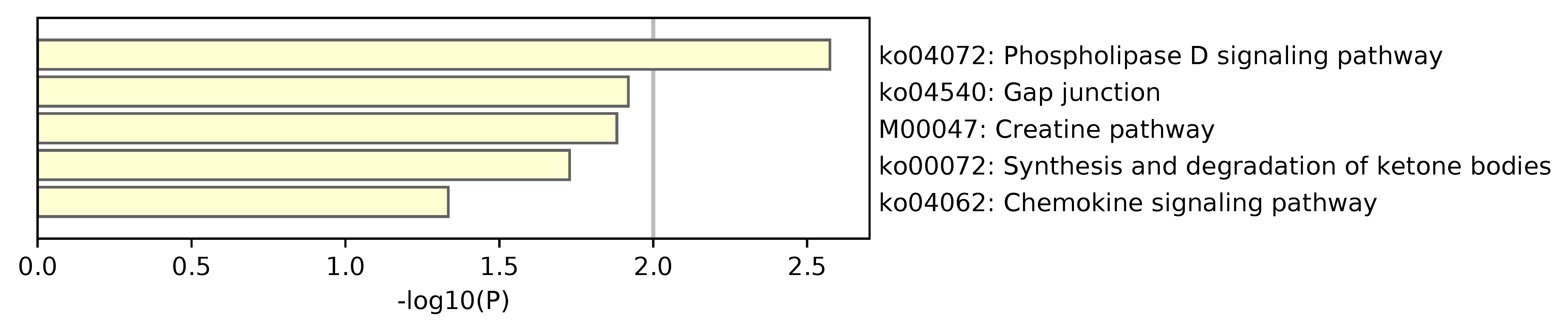

Supplement: Supplementary file 1 [file genes-12-01872-s001.zip › supplementary Figure S4.tiff]
